# Biallelic mutations of *TTC12* and *TTC21B* were identified in Chinese patients with multisystem ciliopathy syndromes

**DOI:** 10.1186/s40246-022-00421-z

**Published:** 2022-10-22

**Authors:** Weicheng Chen, Feifei Wang, Weijia Zeng, Xinyan Zhang, Libing Shen, Yuan Zhang, Xiangyu Zhou

**Affiliations:** 1 Obstetrics and Gynecology Hospital of Fudan University, Pediatric Cardiovascular Center at Children’s Hospital of Fudan University, Fudan University Shanghai Medical College, Shanghai, 200011 China; 2 State Key Lab of Genetic Engineering, School of Life Sciences, Fudan University, Shanghai, 200438 China; 3 Department of Assisted Reproduction, Shanghai First Maternity and Infant Hospital, Tongji University School of Medicine, Shanghai, 201204 China; 4 International Human Phenome Institutes (IHPI), Shanghai, 200433 China; 5 Shanghai, China

**Keywords:** Situs inversus, Heterotaxy, Congenital heart disease, Nephronophthisis, Neonatal cholestasis, Ciliopathy, *TTC21B*, *TTC12*, Recessive mutations

## Abstract

**Background:**

Abnormalities in cilia ultrastructure and function lead to a range of human phenotypes termed ciliopathies. Many tetratricopeptide repeat domain (TTC) family members have been reported to play critical roles in cilium organization and function.

**Results:**

Here, we describe five unrelated family trios with multisystem ciliopathy syndromes, including situs abnormality, complex congenital heart disease, nephronophthisis or neonatal cholestasis. Through whole-exome sequencing and Sanger sequencing confirmation, we identified compound heterozygous mutations of *TTC12* and *TTC21B* in six affected individuals of Chinese origin. These nonsynonymous mutations affected highly conserved residues and were consistently predicted to be pathogenic. Furthermore, ex vivo cDNA amplification demonstrated that homozygous c.1464 + 2 T > C of *TTC12* would cause a whole exon 16 skipping. Both mRNA and protein levels of TTC12 were significantly downregulated in the cells derived from the patient carrying *TTC12* mutation c.1464 + 2 T > C by real-time qPCR and immunofluorescence assays when compared with two healthy controls. Transmission electron microscopy analysis further identified ultrastructural defects of the inner dynein arms in this patient. Finally, the effect of TTC12 deficiency on cardiac LR patterning was recapitulated by employing a morpholino-mediated knockdown of ttc12 in zebrafish.

**Conclusions:**

To the best of our knowledge, this is the first study reporting the association between *TTC12* variants and ciliopathies in a Chinese population. In addition to nephronophthisis and laterality defects, our findings demonstrated that *TTC21B* should also be considered a candidate gene for biliary ciliopathy, such as *TTC26,* which further expands the phenotypic spectrum of TTC21B deficiency in humans.

**Supplementary Information:**

The online version contains supplementary material available at 10.1186/s40246-022-00421-z.

## Background

Ciliopathy is a genetically heterogeneous group of disorders caused by defective or dysfunctional cilia in many organs of the human body that result in variable syndromes, including situs abnormality, respiratory infection, complex CHD, male infertility, nephronophthisis and neonatal cholestasis [1–4]. Accurate positioning of asymmetric organs, known as situs solitus, along the left–right (L–R) axis is essential for their proper function [5]. Defects in LR organization cause laterality disorders, including situs inversus (SI) totalis (SIT) and heterotaxy (Htx) [6]. SIT is a congenital condition in which the organs in the chest and abdomen are arranged in a complete mirror-image reversal of the usual positions; the prevalence of SIT is estimated to range from 1/25000 to 1/8000 [7]. Up to 20% of SIT patients have Kartagener syndrome (KS), which is a triad of nasal polyps, bronchiectasis, SIT, and a subgroup of primary ciliary dyskinesias (PCD) [8]. Approximately 50% of PCD cases displayed laterality defects, mainly SIT. In addition, PCD is often associated with infertility due to immotile sperm in men [9]. Different from SIT, Htx is a condition that involves the internal organs being abnormally arranged but not in a mirror image within the chest and abdomen [10]. Cardiac LR asymmetry disturbance plays significant roles in congenital heart disease (CHD) pathogenesis [4, 11]. Approximately 80% of individuals with Htx have complex congenital heart disease (CHD) [12]. Although SIT patients have a lower risk of CHD (3–9%) than Htx patients, the risk of CHD in SIT is still significantly higher than that in the normal condition, situs solitus (0.6–0.8%) [13]. Nephronophthisis is an autosomal recessive ciliopathy that represents the most frequent genetic cause of end-stage kidney disease in the first three decades of life [14]. Interestingly, many causative genes for nephronophthisis and polycystic kidney, including NPHP2, NPHP3 and PDK2, are also closely related to laterality defects [15–17]. Recent studies found that cholangiocyte cilia are abnormal in biliary atresia (BA), and approximately 20% of BA patients caused by TTC26 demonstrate left–right laterality defects [18, 19], which indicates that cilia participate in the normal development of the intrahepatic biliary system.

Of the many gene families associated with primary cilia function and assembly, the tetratricopeptide repeat domain (TTC) gene family has been extensively illustrated. The cilia protein IFT88 (TTC10) is required for spindle orientation in mitosis [20]. A homozygous null BBS8 (*TTC8*) mutation leads to Bardet-Biedl syndrome (BBS) with randomization of left–right body axis symmetry, a known defect of the nodal cilium [21]. TTC25 deficiency results in primary ciliary dyskinesia with left–right body asymmetry randomization [22]. TTC30A affects tubulin modifications in a model for ciliary chondrodysplasia with polycystic kidney disease [23]. TTC17 regulates actin polymerization and ciliogenesis [24]. *TTC12* loss-of-function mutations cause primary ciliary dyskinesia and unveil distinct dynein assembly mechanisms in motile cilia versus flagella [25]. TTC21B contributes both causal and modifying alleles across the ciliopathy spectrum [26]. In the following, by performing whole-exome sequencing and functional analysis, we identified biallelic mutations of *TTC21B* and *TTC12* that predispose individuals to multisystem ciliopathy syndromes in the Chinese population.

## Results

### Biallelic mutations of TTC21B and TTC12 were identified in Chinese patients with laterality defects, nephronophthisis, or neonatal cholestasis

**Fig. 1:**
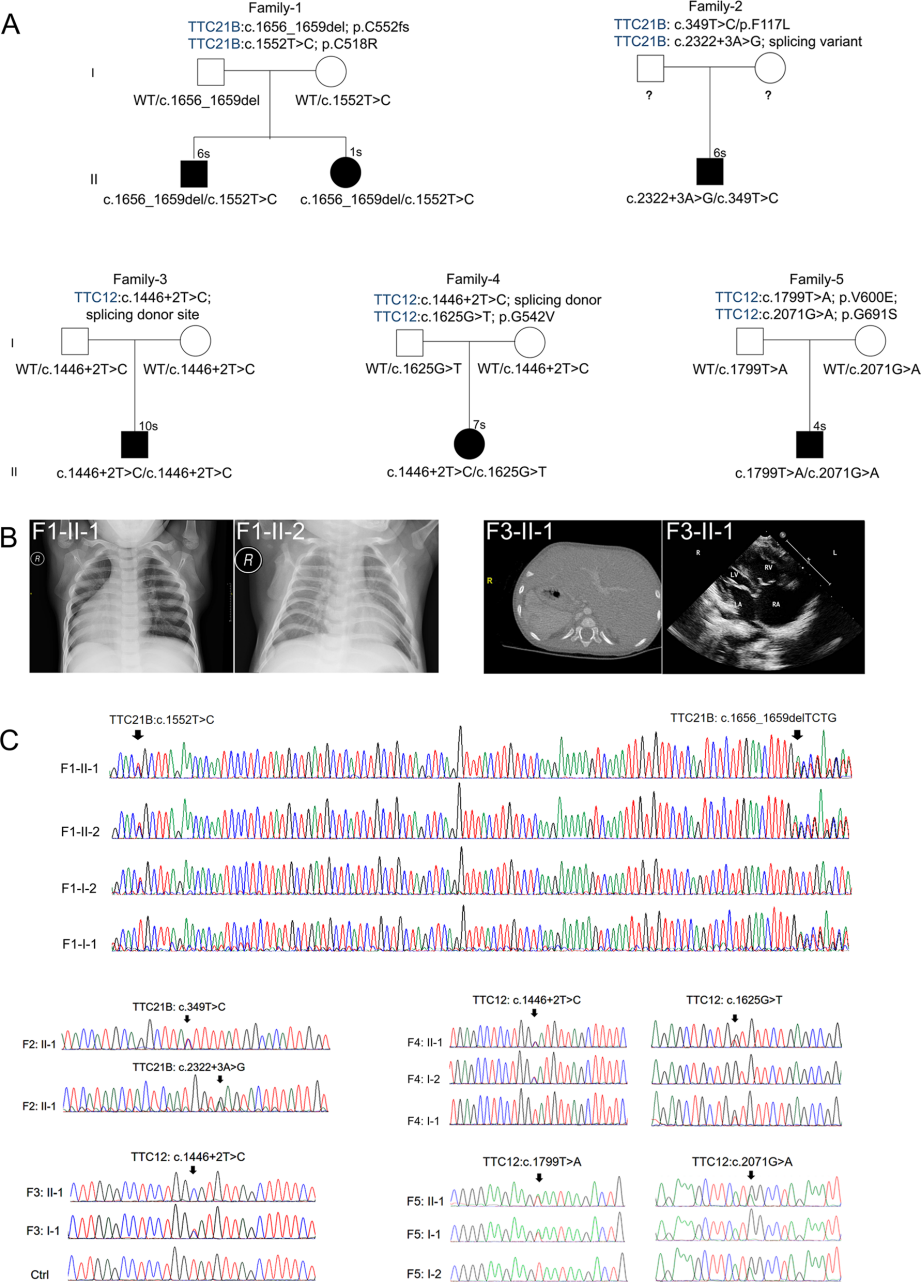
Biallelic TTC21B and TTC12 mutations in five unrelated family trios with multisystem ciliopathy syndromes. **A** Pedigrees of families (F) 1–5 indicating the affected individuals and the segregation of *TTC12* or *TTC21B* heterozygous variants. **B** Representative images of the clinical diagnosis of affected individuals (F1-II-1, F1-II-2, F3-II-1) using chest X-ray imaging, CT scans, or color ultrasound scans as indicated. A mirror-image arrangement of the abdomen is shown in F1-II-1 and F3-II-1. Color-Doppler at the parasternal long axis view showing the parallel relationship of the great arteries and ventriculo–arterial discordance with DORV, ASD and PS in F3-II-1 (right panel). **C** Sanger sequencing of recessive *TTC12* and *TTC21B* variants in six affected individuals and their unaffected parents in five family trios with ciliopathy syndromes. Parent samples were not available for segregation analysis in Family 2

In this study, we describe five unrelated family trios with multisystem ciliopathy syndromes, including situs inversus, complex CHD, nephronophthisis or neonatal cholestasis (Table 1 and Fig. 1A). All the parents of these patients were unaffected and healthy. WES analysis was then performed on affected individuals, and the segregation of candidate variants in their parents was validated by Sanger sequencing except for Family-2. Variant filtering (protein-altering variants, recessive model) was performed according to MAFs under 0.01 in the GnomAD database as previously described [27].

**Table 1:** Clinical phenotypes of six affected individuals carrying biallelic *TTC12* and *TTC21B* mutations in this study

Individual	F1-II-1	F1-II-2	F2-II-1	F3-II-1	F4-II-1	F5-II-1
Mutations	TTC21B:c.1656_1659del	TTC21B:c.1656_1659del	TTC21B:c.2322 + 3A > G	TTC12:c.1464 + 2 T > C	TTC12:c.1464 + 2 T > C	TTC12:c.1799 T > A
	TTC21B:c.1552 T > C	TTC21B:c.1552 T > C	TTC21B:c.349 T > C	homozygous	TTC12:c.1625G > T	TTC12:c.2071G > A
Amino acids	p.C552fs	p.C552fs	Splicing site	Splicing Donor	Splicing Donor	p.V600E
change	p.C518R	p.C518R	p.F117L		p.G542V	p.G691S
MAF in	0.00	0.00	0.0073	0.0033	0.0033	0.000
GnomAD	0.00	0.00	0.00		0.0002	0.0019
SIFT scores	0.001-D	0.001-D	NA	LOF	LOF	0.002-D
	LOF	LOF	0.034-D		0.006-D	0.000-D
Poly-phen2	0.981-D	0.981-D	NA	LOF	LOF	0.991-D
	LOF	LOF	0.034-B		1.000-D	1.000-D
Mutation Taster	0.999-D	0.999-D	NA	LOF	LOF	0.911-D
	LOF	LOF	0.999-D		0.999-D	0.999-D
Age	6 years	1 years	6 years	10 years	2 years	4 years
Sexuality	Male	Female	Male	Male	Female	Male
Respiratory Symptoms	N	N	N	Y	Y	N
Laterality defects	Y	N	N	Y	Y	Y
Complex CHD	N	N	Y	Y	N	N
Nephronophthisis	Y	N	N	N	N	N
Neonatal Cholestasis	N	Y	N	N	N	N
Other Complications	Renal hypertension	Hyperlipidemia, Chronic renal disease	N	N	N	N

*NA* Not available; *CHD* Congenital heart disease; *MAF* Minor allele frequency; *LOF* Loss of function; *D* Damaging and deleterious; *B* Benign

In family-1 (F1), there are two affected individuals. The proband (II-1) was a six-year-old boy who was diagnosed with nephronophthisis-related syndromes and situs inversus totalis. Echocardiography did not detect structural cardiac abnormalities in this boy. His one-year-old sister (II-2) exhibited severe neonatal cholestasis, hyperlipemia and chronic renal disease with elevated serum total and direct bilirubin, gamma-glutamyl transferase, bile acid and transaminase values but had no defects in left–right (LR) patterning (Fig. 1B and Additional file 1: Table S1). Liver biopsy revealed ductular proliferation and cholestatic liver injury with mild fibrosis and inflammatory infiltration. Subsequent WES analysis followed by Sanger sequencing identified compound heterozygous frameshift and missense mutations (c.1656_1659del/p. Cys552fs, c.1552 T > C/p. Cys518Arg) in *TTC21B* (NM_024753)*,* encoding a tetratricopeptide repeat domain 21B protein, in both siblings (Fig. 1C). The frequencies of the minor allele were 0.000 (0/251180) for both paternally derived c.1656_1659del and maternally derived c.1552 T > C in the GnomAD database.

We next identified two heterozygous splice (c.2322 + 3A > G) and missense mutations (c.349 T > C/p. F117 L) of *TTC21B* (NM_024753) in a 6-year-old boy (Family-2-II-1) of Chinese origin by WES analysis (Fig. 1C). Parent samples were not available for segregation analysis. The CT scan did not identify defects in LR patterning. Echocardiography images identified complex CHD, including transposition of great arteries, ventricular septal defect, pulmonary stenosis (PS) and patent ductus arteriosus (PDA) (Additional file 2: Fig. S1A). The MAFs were 0.0073 for c.2322 + 3A > G and 0.000 for p. F117 L in the GnomAD database, respectively.

In Family 3 (F3), the affected individual (II-1) was a 10-year-old boy of Chinese origin (Fig. 1A). A CT scan showed isolated levocardia. Echocardiography images identified complex CHD, including double outlet of right ventricle (DORV), atrial septal defect (ASD), patent foramen ovale (PFO) and pulmonary stenosis (PS) (Fig. 1B and Table 1). WES analysis then identified a homozygous mutation (c.1446 + 2 T > C) of *TTC12* (NM_017868) in this affected individual when compared with a healthy control (Fig. 1C). MAF was 0.00333 in the GnomAD database. This variant was located at the splicing donor site and was predicted to affect exon splicing.

In 2020, a 28-year-old pregnant woman (Family-4:I-2) accessed our online service for genetic counseling at 30 weeks of gestation. Clinical details were obtained from this participant. The fetus was diagnosed as dextroversion without other malformations by ultrasound examination (Additional file 2: Fig. S1B). According to the sequencing report provided by this woman, compound heterozygous mutations (c.1446 + 2 T > C/splicing donor site; c.1625G > T/p. G542 V) of *TTC12* were identified in the fetus through ultrasound-guided amniocentesis, and segregation was further validated by Sanger sequencing of her parents in our study. The MAF was 0.0033 for the paternally derived c.1464 + 2 T > C and 0.0002 for the maternally derived p. G542 V (Table 1).

In Family 5 (F5), the affected individual (II-1) was a 4-year-old boy of Chinese origin with SIT (Fig. 1A). Chest X-ray imaging showed the mirror-image arrangement of abdominal organs but without other malformations. WES then identified compound heterozygous missense mutations (c.1799 T > A/p. V600E; c.2071G > A/p. G691S) of *TTC12* in this affected individual (Fig. 1C). The MAF was 0.000 for the paternally derived p. V606E and 0.0019 for the maternally derived p. G697S (Table 1).

**Fig. 2:**
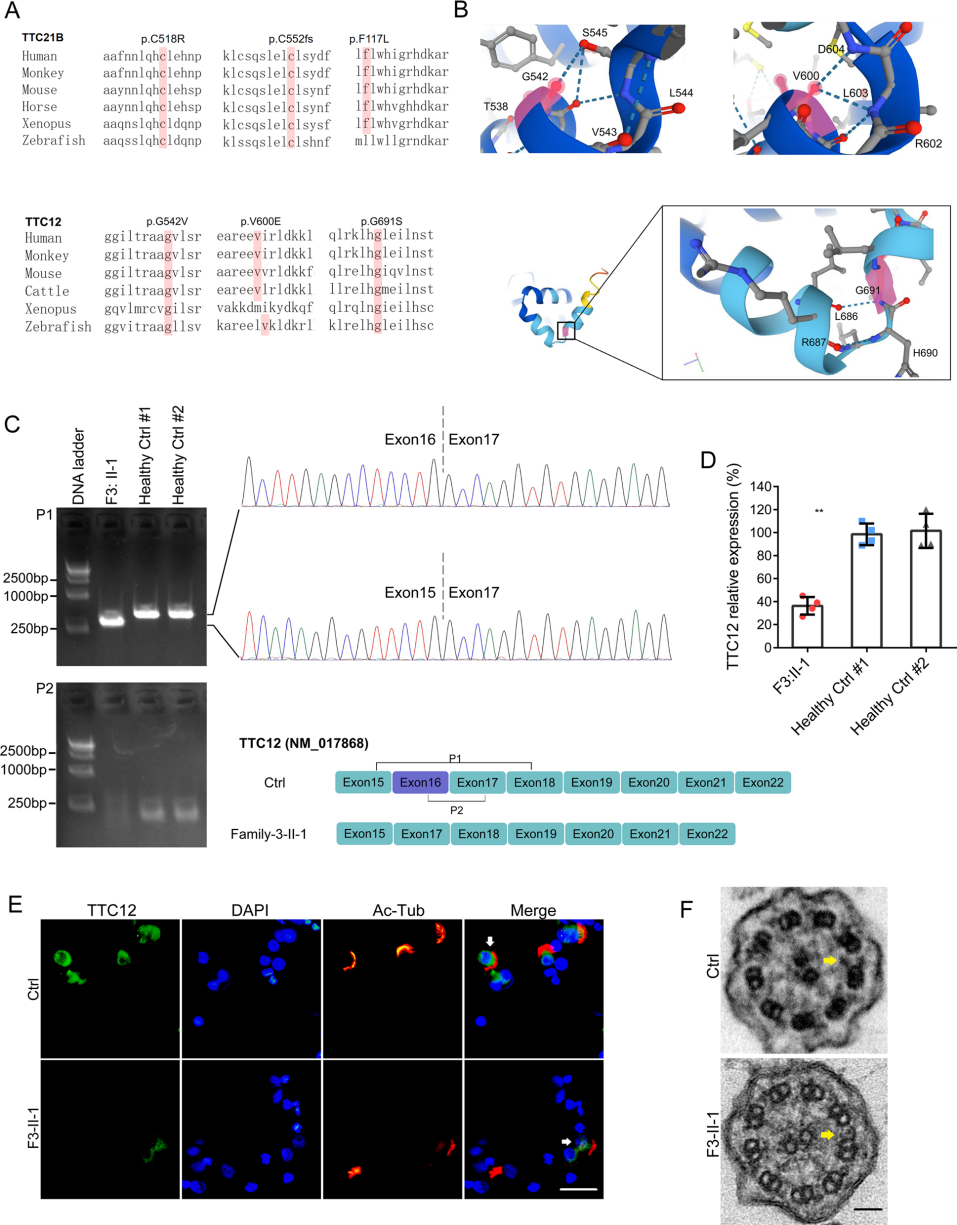
TTC12 c.1446 + 2 T > C causes exon skipping and downregulates its expression, which further leads to ultrastructural defects in IDA. **A** Sequence alignment of *TTC21B* and *TTC12* variants in different species, including zebrafish (*Danio rerio*) and tropical clawed frogs (*Xenopus tropicalis*). **B** AlphaFold protein structure prediction showing the potential effects of amino acid substitutions (G542 V, V600E, and G691S) on hydrogen bonds that might exert a direct influence on the protein structure of *TTC12*. **C** TTC12 exon 16 was entirely skipped in the patient (F3-II-1) carrying c.1446 + 2 T > C of TTC12 (NM_017868) by cDNA amplification followed by gel extraction and Sanger sequencing. cDNA derived from fresh nasal samples of this patient and two healthy volunteers was amplified. Representative images from three independent experiments are displayed. **D** mRNA levels of TTC12 were downregulated in the patient carrying c.1446 + 2 T > C by real-time quantitative PCR. ^**^p value < 0.01, (two-tailed Student’s *t* test; n = 4). **E** Immunofluorescence staining with anti-TTC12 antibody showed the decreased signal intensity of TTC12 in the cytoplasm of cells derived from the patients (F3-II-1) when compared with healthy controls (Ctrl). Scale bar, 10 µm. **F** Representative images of transmission electron micrographs (TEM) of cross sections of ciliary axonemes from the patients (F3-II-1) and a healthy control (Ctrl). Distal absence of IDA is indicated by the yellow arrow. Scale bar, 50 nm

Subsequent sequence alignment analysis indicated that all these nonsynonymous mutations of *TTC12* and *TTC21B* from WES analysis were highly conserved in different species, including Zebrafish (*Danio rerio*) and Tropical clawed frog (*Xenopus tropicalis*) (Fig. 2A), and were consistently predicted to be pathogenic by Poly-phen2, Mutationtaster and SIFT (Table 1). According to the AlphaFold protein structure database [28], the amino acid substitutions in *TTC12,* including G542 V, V600E and G691S, were predicted to destroy hydrogen bonds that potentially exert a direct influence on protein structure (Fig. 2B).

### *Skipped exon and downregulated expression of TTC12 in the patient carrying homozygous c.1446* + *2 T* > *C*

To investigate the biological consequences of the splicing donor site variant c.1464 + 2 T > C in TTC12, we performed real-time PCR using cDNAs derived from nasal tissue samples of the patient (F3-II-1) and two healthy controls. A smaller PCR band was generated in this patient by cDNA amplification (Fig. 2C). Sanger sequencing further demonstrated that exon 16 of TTC12 was entirely skipped in the smaller amplification product (Fig. 2C). Subsequent real-time qPCR (Fig. 2D) and immunofluorescence staining (Fig. 2E) demonstrated that TTC12 expression was significantly downregulated in the nasal mucosa of the patient carrying TTC12 mutations compared with the healthy controls. A previous study reported that the inner dynein arms (IDAs) were absent in respiratory cilia in affected individuals carrying *TTC12* loss-of-function mutations [26]. We next assessed the effect of *TTC12* c.1446 + 2 T > C on the ultrastructure of cilia in the nasal mucosa by TEM. Overall, we examined > 50 ciliary cross sections without bias toward measurements in any particular ciliary region. Although the outer dynein arms (ODA) were not affected by TTC12 mutations, we identified obvious IDA defects in the patient (F3:II-1) carrying c.1446 + 2 T > C of *TTC12* when compared with the healthy control (Fig. 2F).

### TTC12 knockdown leads to defective cardiac LR patterning in zebrafish.

**Fig. 3:**
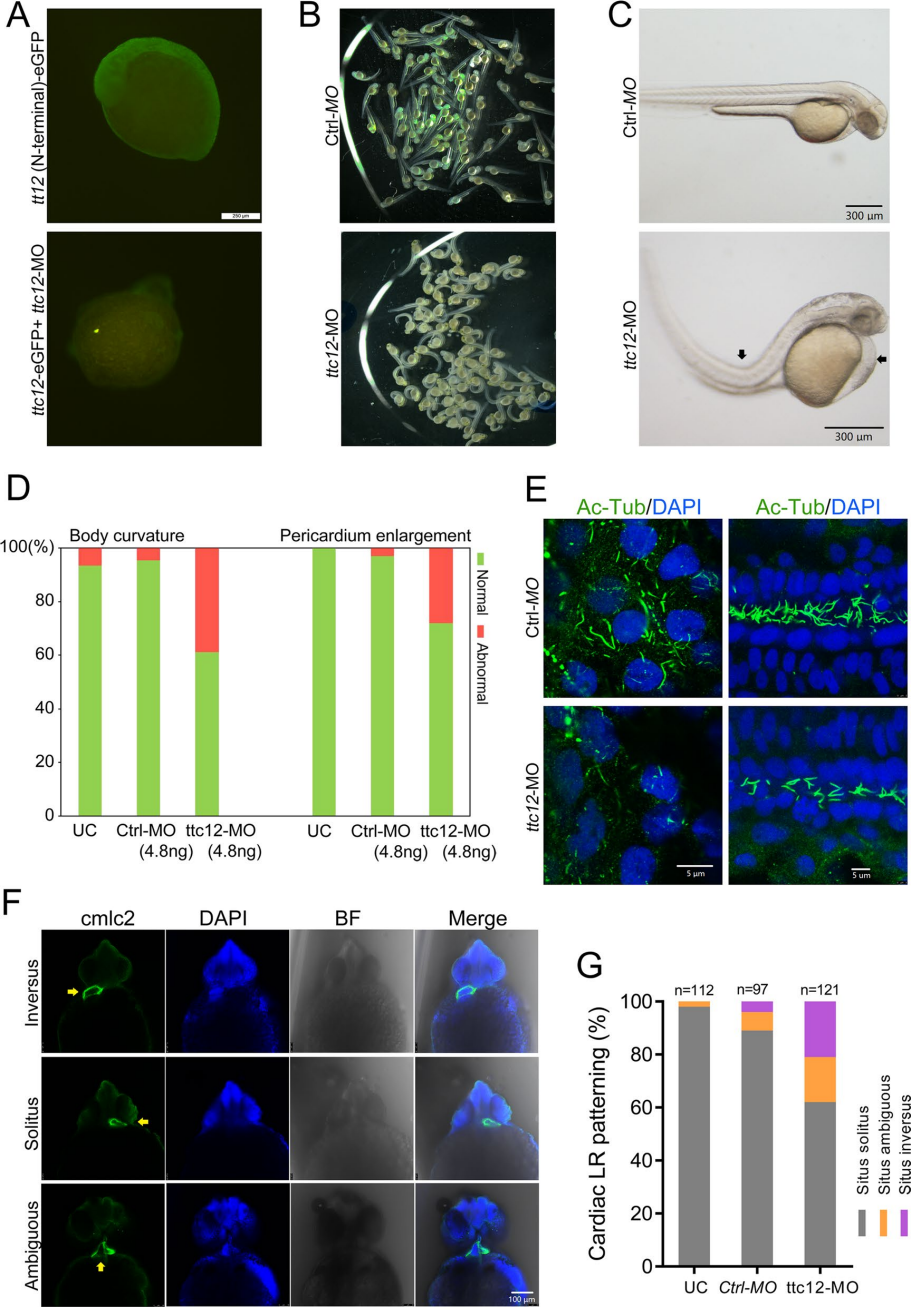
TTC12 knockdown causes defective LR patterning in zebrafish embryos. **A** Efficiency of ttc12-MO is evaluated by simultaneous coinjection of ttc12 (N-terminal)-eGFP mRNA that effectively abrogates GFP signals in embryos at 16 hpf. Scale bar, 250 μm. **B** An overview of the morphology of zebrafish embryos injected with Ctrl-MO or ttc12-MO. **C** A representative image of body curvature and pericardium enlargement in ttc12-MO-treated embryos is shown. Scale bar, 300 μm. **D** Percentage of body curvature and pericardium enlargement in ttc12-MO- or Ctrl-MO-treated embryos as indicated. **E** A mild reduction in cilia density was identified in the tail fin (right panels) and pronephros (left panels) of ttc12-MO-treated embryos compared with Ctrls. A representative image is shown. Scale bar, 5 μm. **F** Representative images of whole-mount immunofluorescence showing inversus, solitus or ambiguous situs in zebrafish embryos by staining cardiac myosin light chain-2 (cmlc2) antibody and DAPI using a confocal microscope at 48 h postfertilization. Scale bar, 100 μm. **G** Percentage of embryos with situs solitus, situs ambiguous, and situs inversus in the uninjected control (UC) and embryos injected with *ttc12*-MO or Ctrl-MO

Although TTC12 is classified as a new causative gene for PCD, its role in LR patterning establishment has not yet been investigated. Therefore, we next employed morpholino (MO) antisense oligonucleotide-mediated knockdown of ttc12 in zebrafish. To test the efficiency of ttc12-MO, a partial fragment of the N-terminal region of the zebrafsh ttc12 open reading frame, including the start codon, was cloned into the pcDNA3.1-eGFP vector. GFP signals could be detected in embryos at 16 hpf after injection of ttc12 (N-terminal)-eGFP mRNA. Simultaneous coinjection of TTC12-MO effectively abrogated GFP expression (Fig. 3A). Compared with uninjected controls and those administered 5-base mismatched Ctrl-MO, we observed multiple ciliary-associated phenotypes, including curved body and pericardial enlargement in ttc12-silenced embryos at 48 hpf (Fig. 3B–D). Although cilia length was not significantly disturbed, we observed a mild reduction in cilia density in the pronephros and tail fin of ttc12-silenced embryos when compared with Ctrl-MO (Fig. 3E). Furthermore, we found that 38.4% of ttc12-silenced embryos displayed abnormal cardiac LR patterning and heart-looping formation (Fig. 3F-G) according to cardiac myosin light chain-2 (cmlc2) immunofluorescence staining at 48 h postfertilization. These data suggested that *ttc12* was also required for normal cardiac left–right organization in zebrafish.

## Discussion

In this study, we identified biallelic mutations in *TTC12* in three unrelated Chinese families with multisystem ciliopathy syndromes, providing genetic evidence to support the critical roles of *TTC12* in cilia assembly or function. To the best of our knowledge, this is the second report that reveals the association between *TTC12* and human ciliopathy. The obtained genetic evidence from different ethnicities [24] and ours are adequate to conclude a causal link for this correlation. Furthermore, the LR laterality defects, a typical syndrome of PCD, in the patient carrying the *TTC12* mutation were exactly recapitulated in zebrafish by employing a morpholino -mediated knockdown of ttc12.

Although *TTC21B* has been intensively illustrated in numerous individuals with kidney diseases, including nephronophthisis type 12 and glomerular and cystic kidney diseases [26, 29–32], our results found that *TTC21B* mutations were also associated with biliary ciliopathy, such as *TTC26* mutations. Although we did not generate experimental data to support a connection between neonatal cholestasis and *TTC21B* mutation in our study, several studies have established that neonatal cholestasis is a newly characterized hepatic ciliopathy, termed biliary ciliopathy. Loss of cilia and mutations in cilia-related genes would cause neonatal cholestasis [19, 33, 34]. *TTC26* is a biliary ciliopathy human disease gene and all seven patients carrying recessive *TTC26* mutations (7/7) presented with neonatal cholestasis and liver impairment [19]. Liver biopsy further revealed liver cirrhosis and ductular proliferation in five patients carrying *TTC26* mutations, which is basically the same as the clinical symptoms of the individual (F1-II-2) carrying *TTC21B* mutations in our study. Both TTC21B and TTC26 contain tetratricopeptide repeat (TPR) domains and are involved in retrograde intraflagellar transport in cilia. In addition to classical ciliopathy phenotypes, a 47-year-old male carrying *TTC21B* mutations also presented with grade B biliary cirrhosis [35]. In mice, perinatal deletion of Ttc21b (also termed as Thm1) resulted in disorganized and expanded biliary regions, biliary fibrosis, increased serum bile acids, and a shortened primary cilium on epithelial cells [36]. These findings provide additional evidence to support the connection between biliary ciliopathy and *TTC21B* mutation, which at least in part expands the phenotypic spectrum associated with mutations of *TTC21B* in humans.

The main concern in the present study is marked phenotypic heterogeneity in two siblings carrying the same recessive mutations of *TTC21B*. Phenotypic heterogeneity is a complex problem in current ciliopathy studies involving environmental and epigenetic factors, oligogenic inheritance and modifier genes. For example, a previous study reported that eight unrelated individuals from different ethnicities carrying the same *TTC26* mutations (c.695A > G, p.Asn232Ser) presented with highly variable clinical symptoms [37]. Therefore, we postulated that the difference in genetic background of the two individuals might be the reason. To explore the possible effector, we next performed genome-wide copy number variation (CNV) analysis that identified a de novo duplication at chromosome 16 (16p13.1) in the patient (II-1) but not in his sister (II-2) in Family 1 (Additional file 3, 4 and 5: Fig. S2, Table S2, Supplementary Methods). Chromosomal alterations in 16p13.11 have been implicated in many human diseases, including congenital anomalies of the kidney and urinary tract and cardiac abnormalities [38, 39]. A recent study found that gain of copy at chromosome 16 could push the genetic background closer to the threshold for severe manifestation and therefore require a lesser contribution from other hits [40]. Thus, de novo 16p13.11 duplications might act as a genetic modifier to alter the phenotypic consequence of these rare mutations. Accurate genetic diagnosis requires complete evaluation of the genetic background even after a candidate disease-associated variant is identified.

In addition to genetic background, epigenetic factors may also play a role in phenotypic heterogeneity. Phenotypic discordance was frequently observed in numerous monozygotic twins [41]. For example, we once located monozygotic twins, and the 4-year-old boy displayed isolated dextrocardia accompanied by an atrial septal defect. The identical twin brother exhibited congenital nystagmus, but no other developmental malformations. The intrauterine environment and genetic background are supposed to be the same in monozygotic twins. There are several possible explanations for these observations, but one is the existence of epigenetic differences. In fact, a previous study found that older monozygous twins exhibited remarkable differences in their overall content and genomic distribution of 5-methylcytosine DNA and histone acetylation, affecting their gene expression portrait [42]. A recent study by Robson A et al., found that histone 2B monoubiquitination (H2Bub1) marks are enriched on the cilia transcription factor in a tissue-specific manner and epigenetic control of cilia motility mediated by HuBub1 regulates heart development [43]. In addition, Wang L et al., uncovers dynamic DNA methylation as an epigenetic mechanism to regulate lefty2 expression and control left–right determination during early embryogenesis in vertebrates [44]. These findings suggested that tissue-specific regulation mediated by epigenetic factors might be the main cause of differences. Epigenetic factors can act as modifiers to influence the clinical manifestations in individuals carrying the same pathogenic mutations. Therefore, deeply dissecting the role of these possible effectors on phenotypic heterogeneity would be an avenue for further understanding these observations.

TTC21B is localized to the cilium axoneme and plays a role in retrograde intraflagellar transport [45]. Previous studies [29–32] and ours identified *TTC21B* mutations in several patients with situs inversus. It is well known that approximately 50% of PCD patients have SIT. However, we did not identify the typical PCD symptoms, including sinusitis, otitis media and bronchitis, in these patients, the lack of which is further supported by the lung CT scan assessment, measurement of nasal nitric oxide levels, and patient history. Although we could not exactly explain the mechanism for the apparent absence of respiratory symptoms in these patients carrying *TTC21B* mutations, these observations demonstrate that SIT might also occur in a non-PCD-dependent manner. In contrast to *TTC12* expression, which is restricted to spermatocytes and respiratory ciliated cells, *TTC21B* was universally expressed in most tissues but possessed relatively lower expression levels in spermatocytes and respiratory ciliated cells (Additional file 6: Fig. S3). It seems that tissue specificity of gene expression provides, at least in part, sensitivity to determine the possible clinical symptoms.

## Conclusion

To the best of our knowledge, we provide the first genetic evidence to support the correlation between *TTC12* variants and ciliopathies in the Chinese population. Meanwhile, our study further expands the phenotypic spectrum of TTC21B deficiency.

## Methods

### Subjects

In this study, affected individuals were diagnosed using X-ray, computed tomography (CT) and color ultrasonic scans at the Pediatric Cardiovascular Center of the Children's Hospital affiliated with Fudan University, Shanghai, China. Detailed clinical features of the patients are provided in Table 1. For studies of affected individuals and their families, written informed consent was obtained from all participants prior to the start of the blood drawing and nasal biopsy. All procedures in the study were approved by the Medical Ethics Committee of the Children’s Hospital of Fudan University (2016–079) (Shanghai, China).

### Genetic analysis

Whole-exome sequencing (WES) was performed by utilizing the SureSelect human all exon platform (v.6; Agilent Technologies, Santa Clara, CA, USA). The Genome Analysis Toolkit software package (GATK4.1.2.0) was used for the detection of single-nucleotide variants and indels. Variant filtering was performed according to minor allele frequencies (MAF) under 0.01 in the GnomAD database (http://gnomad.broadinstitute.org/) as previously described [27], given the rarity of situs abnormalities and associations with protein-altering variants (missense, nonsense, and splice-site variants as well as coding indels). Candidate recessive variants were then maintained for subsequent analysis. The effects of the identified variants were assessed using SIFT (http://sift.jcvi.org), PolyPhen-2 (genetics.bwh.harvard.edu/pph2), and Mutationtaster (http://mutationtaster.org). Conservation analysis was performed using Multiple Sequence Alignment ClustalW2 (ebi.ac.uk/Tools/msa/clustalw2). Variants in *TTC12* and *TTC21B* were further confirmed by Sanger sequencing, and the sequences of the primers are provided in Additional file 7: Table S3.

### cDNA analysis and RT‒qPCR

Total RNA was isolated from fresh nasal tissue samples of the patients and healthy volunteers using the RNAsimple Total RNA kit (DP419; TIANGEN BIOTECH, Beijing, China), followed by first-strand cDNA synthesis (KR106; TIANGEN). cDNA was then amplified by polymerase chain reaction (PCR) with specific TTC12 primers, followed by gel extraction (DP209; TIANGEN) and Sanger sequencing. RT‒qPCR was performed using FastFire qPCR PreMix (SYBR Green) (FP207, TIANGEN) on an ABI StepOnePlus instrument. The delta-delta-Ct (ddCt) algorithm was utilized to analyze the relative changes in gene expression by using GAPDH as the internal reference (housekeeping gene). Primers were designed using the Primer3Plus software to span exon-exon junctions and provided in Additional file 8: Table S4.

### Immunofluorescence

Nasal tissues of healthy individuals and the patient carrying TTC12 mutations were seeded on glass cover slips and grown in the presence of 10% FBS under identical culture conditions as previously described [46]. Then, the tissues were fixed and permeabilized for 10 min using 4% PFA and 0.8% Triton X-100. After blocking (10% normal goat serum [NGS]), slides were incubated with the following primary antibodies: anti-TTC12 (Santa Cruz Biotechnology, sc-390229, 1:100 dilution) and anti-acetyl-α-tubulin (Cell Signaling Technology, #5335, 1:500 dilution). The slides were then incubated with the secondary antibody (Goat anti-rabbit or -mouse IgG, Alexa Fluor 488 or 594; Thermo Fisher Scientific, 1:1000 dilution) for 2 h, followed by staining with DAPI (Thermo Fisher Scientific)/PBS for 6 min. Confocal imaging was performed using an SP8 system (Leica), and images were processed using the Leica AF software suite.

### Ttc12 knockdown in Zebrafish

Studies were undertaken using AB strain zebrafish (*Danio rerio*) under standard conditions as previously described [47]. We injected a 2.3-nL mixture of *ttc12*-MO (antisense to the zebrafish *ttc12* start codon) or specific 5-base mismatched oligos (Ctrl-MO) into each one- to two-cell-stage zebrafish embryo. Zebrafish *ttc12*-MO was purchased from Gene Tools, LLC (Philomath, OR, USA), with the following sequence: ttc12-MO, 5ʹ- AGACATGGTTGAAACAGAGTTCATA-3ʹ; Ctrl-MO, 5ʹ-AGAgATcGTTcAAACAGAcTTgATA-3ʹ. At 48 hpf, images of the harvested embryos were taken, and their morphologies were analyzed.

### Whole-mount immunohistochemistry

Zebrafish embryos at 48 hpf were fixed in 4% paraformaldehyde (PFA) overnight at 4 °C and stored in 100% MeOH at − 20 °C. Prior to the assay, MeOH was removed followed by PBST washing. Embryos were permeabilized in prechilled acetone at − 20 °C for 10 min and then incubated with blocking buffer (10% normal goat serum [NGS]/PBST) at 4 °C for 3 h, followed by transfer to 1% NGS/PBST containing one of the primary antibodies for incubation at 4 °C for 2 days. We used anti-cmlc2 (1:500 dilution, GTX128346; GeneTex, Irvine, CA, USA) according to the manufacturer’s instructions. Embryos were incubated with the secondary antibody (Thermo Fisher Scientific, Alexa 488 anti-rabbit, 1:1000 dilution) for 2 h at room temperature, followed by staining with DAPI.

### Statistics

All data are presented as the mean ± S.D. A student’s two-tailed unpaired t test was used for comparisons of means of quantitative data between groups using GraphPad Prism software (v.5.01; GraphPad Software, CA, USA). Differences were considered significant at *p* < 0.05.

## Supplementary Information

**Additional file 1: Table S1.** The hepatic manifestations of the patient (Family-1-II-2) carrying *TTC21B* mutations at the age of 2 months

**Additional file 2: Fig S1.** (A) Echocardiography shows transposition of great arteries and ventricular septal defect in the patient JM0087 (F-2: II-1). (B) Chest X-ray shows dextrocardia in the patient JM0529 (F-4:II-1) at the age of 2 years

**Additional file 3: Fig. S2.** Exome-based copy number alteration detection was performed followed by MLPA confirmation. (A-B) CNV analysis identified 16p13.11 microduplication (chr16: 14.82-15.12, 300kb) in the F1-II-1 (607P) but not in F1-II-2 (607S). The individual 692F served as a control. (C) MLPA confirmation of the gain of copy in *PDXDC1*, located in the 16p13.11 region, that identified from CNV analysis in the 607-P when compared with 607-S. Three independent probes were designed for each targeted gene as indicated. GAPDH served as the internal reference

**Additional file 4: Table S2.** Probes sequences targeting *NUP188* and *PDXDC1* in MLPA. GAPDH served as a internal control

**Additional file 5:** Supplementary Methods

**Additional file 6: Fig. S3.** Different from TTC12 expression that restricted to spermatocytes and respiratory ciliated cells (upper), TTC21B was universally expressed in most of tissues but possess relative lower expression levels in spermatocytes and respiratory ciliated cells (bottom) according to Human Protein Atlas Database (www.proteinatlas.org)

**Additional file 7: Table S3.** Primers used for Sanger sequencing for *TTC21B* and *TTC12* variants that identified from WES analyzes

**Additional file 8: Table S4.** Primers used for real-time PCR and cDNA amplification of *TTC12*

## Data Availability

All raw data that support the findings of this study are available from the corresponding authors upon reasonable request.

## Abbreviations

TTC: Tetratricopeptide
CHD: Congenital heart disease
WES: Whole-exome sequencing
TEM: Transmission electron microscopy
IDA: Inner dynein arms
MO: Morpholino
SIT: Situs inversus totalis
Htx: Heterotaxy
PCD: Primary ciliary dyskinesias
CNV: Copy number variation
cmcl2: Cardiac myosin light chain-2
