# Supplementary material for: Biallelic mutations of TTC12 and TTC21B were identified in Chinese patients with multisystem ciliopathy syndromes
Source: Hum Genomics. 2022 Oct 22;16:48. doi: 10.1186/s40246-022-00421-z (PMC9587637; doi:10.1186/s40246-022-00421-z)
Supplement: Supplementary file 1 — Additional file 1: Table S1. The hepatic manifestations of the patient (Family-1-II-2) carrying TTC21B mutations at the age of 2 months [file 40246_2022_421_MOESM1_ESM.pdf]

Table S1. The hepatic manifestations of the patient (Family-1-II-2) at the age of 2 months

Family-1 (II-2)	
AST (5-30 U/L)	↑436
ALT (5-30 U/L)	↑632
GGT (5-36 U/L)	↑89
ALP (50-100 U/L)	↑583
Direct bilirubin (0-6 μmol/L)	↑165.2
Total bilirubin (2-20 μmol/L)	↑307.1
Albumin (35-50 g/L)	33.2
