# Supplementary figures and images for: Biallelic mutations of TTC12 and TTC21B were identified in Chinese patients with multisystem ciliopathy syndromes
Source: Hum Genomics. 2022 Oct 22;16:48. doi: 10.1186/s40246-022-00421-z (PMC9587637; doi:10.1186/s40246-022-00421-z)

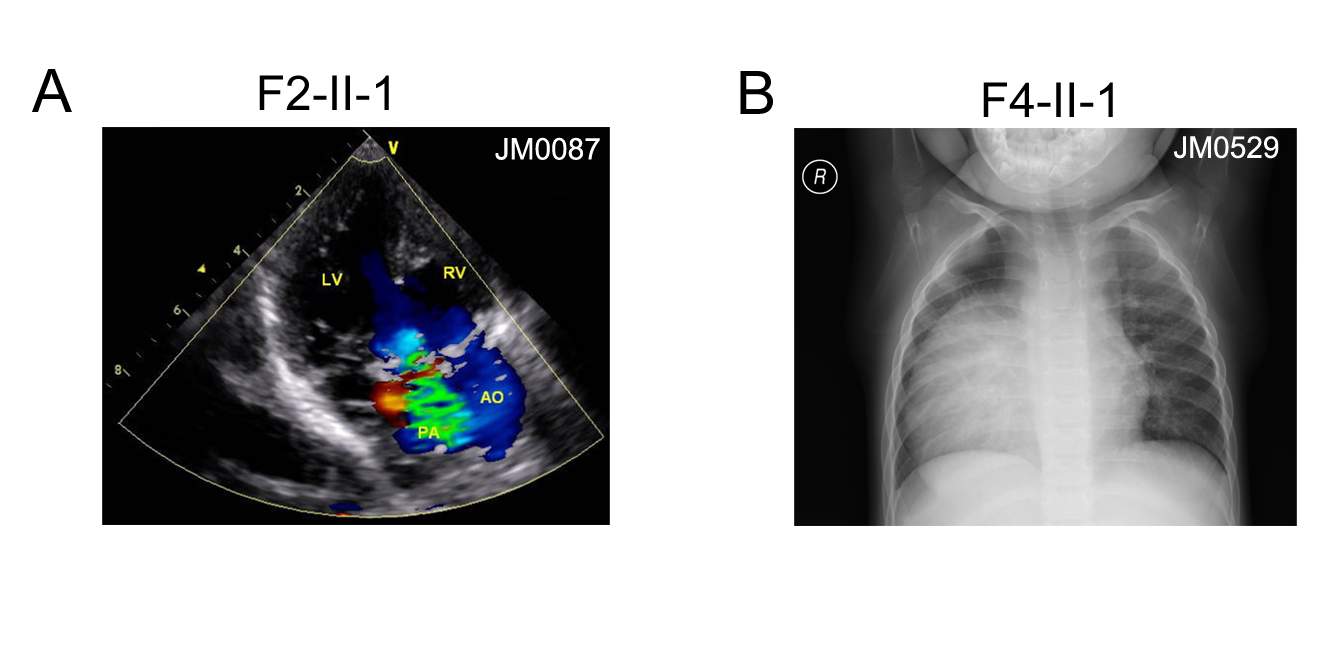

Supplement: Supplementary file 2 — Additional file 2: Fig S1. (A) Echocardiography shows transposition of great arteries and ventricular septal defect in the patient JM0087 (F-2: II-1). (B) Chest X-ray shows dextrocardia in the patient JM0529 (F-4:II-1) at the age of 2 years [file 40246_2022_421_MOESM2_ESM.tif]

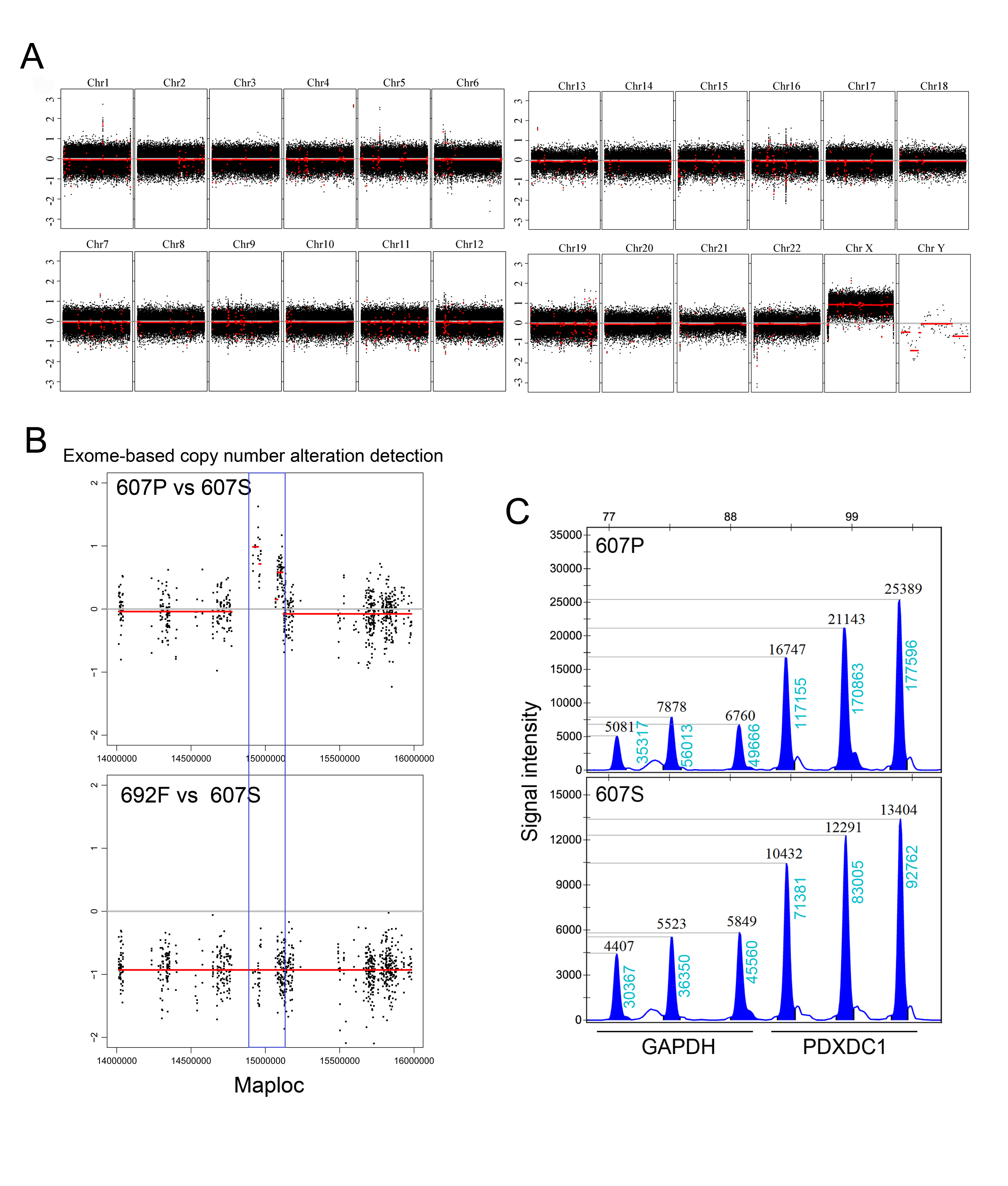

Supplement: Supplementary file 3 — Additional file 3: Fig. S2. Exome-based copy number alteration detection was performed followed by MLPA confirmation. (A-B) CNV analysis identified 16p13.11 microduplication (chr16: 14.82-15.12, 300kb) in the F1-II-1 (607P) but not in F1-II-2 (607S). The individual 692F served as a control. (C) MLPA confirmation of the gain of copy in PDXDC1, located in the 16p13.11 region, that identified from CNV analysis in the 607-P when compared with 607-S. Three independent probes were designed for each targeted gene as indicated. GAPDH served as the internal reference [file 40246_2022_421_MOESM3_ESM.tif]

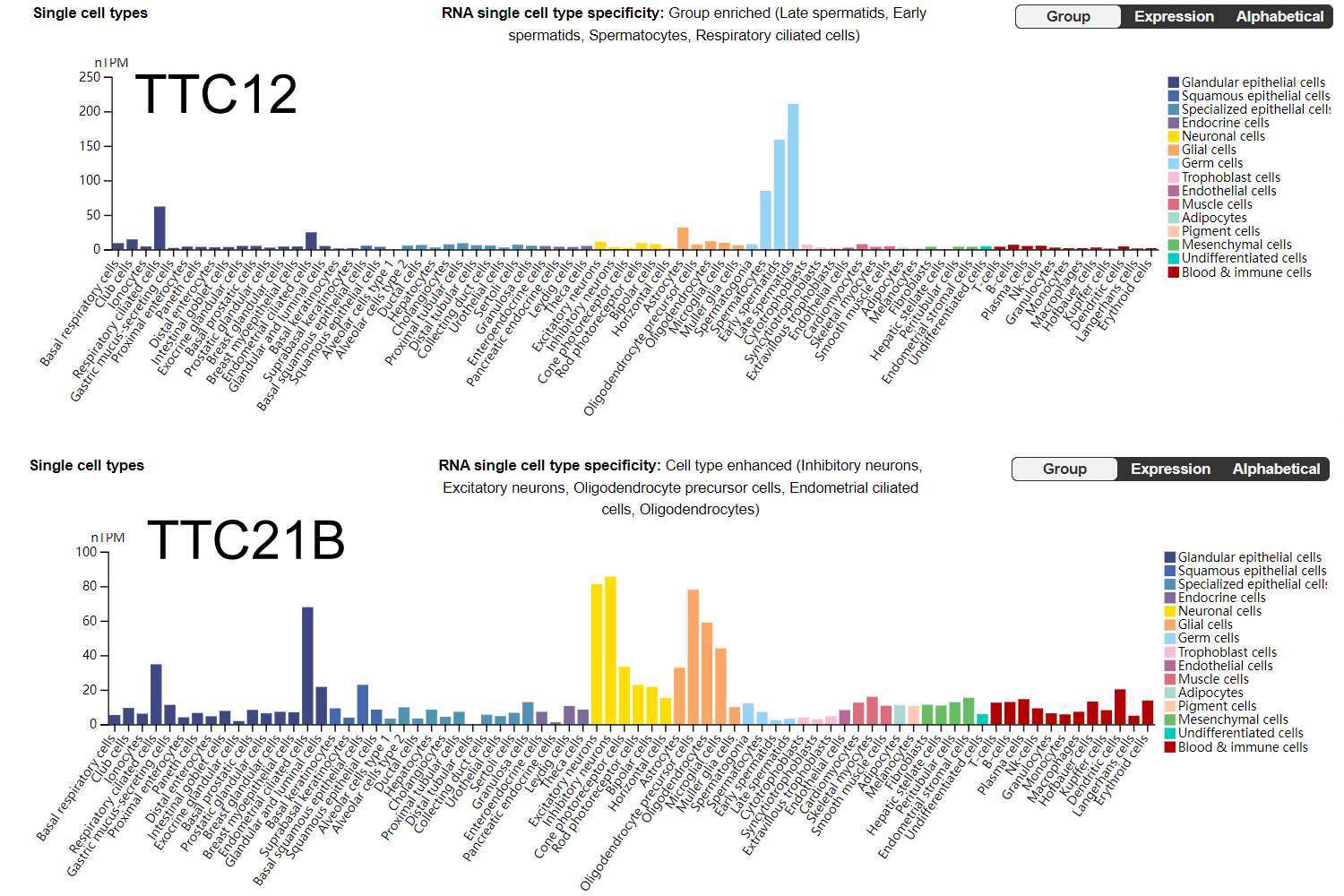

Supplement: Supplementary file 6 — Additional file 6: Fig. S3. Different from TTC12 expression that restricted to spermatocytes and respiratory ciliated cells (upper), TTC21B was universally expressed in most of tissues but possess relative lower expression levels in spermatocytes and respiratory ciliated cells (bottom) according to Human Protein Atlas Database (www.proteinatlas.org) [file 40246_2022_421_MOESM6_ESM.tif]
