# Supplementary material for: Biallelic mutations of TTC12 and TTC21B were identified in Chinese patients with multisystem ciliopathy syndromes
Source: Hum Genomics. 2022 Oct 22;16:48. doi: 10.1186/s40246-022-00421-z (PMC9587637; doi:10.1186/s40246-022-00421-z)
Supplement: Supplementary file 4 — Additional file 4: Table S2. Probes sequences targeting NUP188 and PDXDC1 in MLPA. GAPDH served as a internal control [file 40246_2022_421_MOESM4_ESM.pdf]

| Primer | Sequences (5'to3') | Length (nt) | |
|---|---|---|---|
| NUP188-LPO-1: | GGGTTCCCTAAGGGTTGGAGGATTTGGTAGTAGGCAGACCAATAGGCACCTGG | 53 | 110 |
| NUP188-RPO-1: | TGGATGAGACTATGGATCCTTTTGTAGATCGGATTCTAGATTGGATCTTGCTGGCAC | 57 | |
| NUP188-LPO-2 | GGGTTCCCTAAGGGTTGGACCAGCTGCTGATCAAGACAGTGAAACTGGCATTCTC | 55 | 115 |
| NUP188-RPO-2 | CGTCACCAACAATGTTATTCGGCTGAAACCTCCTTCTTCTAGATTGGATCTTGCTGGCAC | 60 | |
| NUP188-LPO-3 | GGGTTCCCTAAGGGTTGGATGTGCTCAGGTCCTCACGCTGCAGACGCCCCCTAGAGGA | 58 | 120 |
| NUP188-RPO-3 | ACTTTCCTTCCTTTCCAGCATTCCCCACAGCACTGCCGGTCTAGATTGGATCTTGCTGGCAC | 62 | |
| PDXDC1-LPO-1: | GGGTTCCCTAAGGGTTGGATCCGGTGTTTAAAGCCGTCCCAGTGC | 45 | 95 |
| PDXDC1-RPO-1: | CCAACATGACACCTTCAGGAGTCGGCCTCTAGATTGGATCTTGCTGGCAC | 50 | |
| PDXDC1-LPO-2 | GGGTTCCCTAAGGGTTGGAGTTGCCTTCCTGGAGAAACTGATTAAAGA | 48 | 100 |
| PDXDC1-RPO-2 | TGATATAGAGCGAGGAAGACTGCCCCTGTTCTAGATTGGATCTTGCTGGCAC | 52 | |
| PDXDC1-LPO-3 | GGGTTCCCTAAGGGTTGGATAGGACACACAGACAAGATTGGGAGATTGAAA | 51 | 105 |
| PDXDC1-RPO-3 | GAACTCTGTGAGCAGTATGGCATATGGCTTCTCTAGATTGGATCTTGCTGGCAC | 54 | |
| IC-LPO1-1 | GGGTTCCCTAAGGGTTGGAAGGAAATGAATGGGCAGCC | 38 | 80 |
| IC–RPO1-1 | PO4-GTTAGGAAAGCCTGCCGGTTCTAGATTGGATCTTGCTGGCAC | 42 | |
| IC-LPO2-1 | GGGTTCCCTAAGGGTTGGATGCTCCCACTCCTGATTTCTG | 40 | 85 |
| IC–RPO2-1 | PO4-GAAAAGAGCTAGGAAGGACAGGTCTAGATTGGATCTTGCTGGCAC | 45 | |
| IC-LPO3-1 | GGGTTCCCTAAGGGTTGGAGGTTCATAACTGTCTGCTTCTCTG | 43 | 90 |
| IC–RPO3-1 | PO4-CTGTAGGCTCATTTGCAGGGGGGATCTAGATTGGATCTTGCTGGCAC | 47 | |
