# Supplementary material for: Biallelic mutations of TTC12 and TTC21B were identified in Chinese patients with multisystem ciliopathy syndromes
Source: Hum Genomics. 2022 Oct 22;16:48. doi: 10.1186/s40246-022-00421-z (PMC9587637; doi:10.1186/s40246-022-00421-z)
Supplement: Supplementary file 5 — Additional file 5: Supplementary Methods [file 40246_2022_421_MOESM5_ESM.docx]

**SUPPLEMENTARY FIGURE AND TABLE LEGENDS**

**Figure S1.** (A) Echocardiography shows transposition of great arteries and ventricular septal defect in the patient JM0087 (F-2: II-1). (B) Chest X-ray shows dextrocardia in the patient JM0529 (F-4:II-1) at the age of 2.

**Figure S2**. Exome-based copy number alteration detection was performed followed by MLPA confirmation. (A-B) CNV analysis identified 16p13.11 microduplication (chr16: 14.82-15.12, 300kb) in the F1-II-1 (607P) but not in F1-II-2 (607S). The individual 692F served as a control. (C) MLPA confirmation of the gain of copy in PDXDC1, located in the 16p13.11 region, that identified from CNV analysis in the 607-P when compared with 607-S. Three independent probes were designed for each targeted gene as indicated. GAPDH served as the internal reference.

**Figure S3**. Different from TTC12 expression that restricted to spermatocytes and respiratory ciliated cells (upper), TTC21B was universally expressed in most of tissues but possess relative lower expression levels in spermatocytes and respiratory ciliated cells (bottom).

**Table S1**. The hepatic manifestations of the patient (Family-1-II-2) carrying *TTC21B* mutations at the age of 2 months

**Table S2**. Primers used for Sanger sequencing for *TTC21B* and *TTC12* variants that identified from WES analyzes.

**Table S3**. Primers used for real-time PCR and cDNA amplification of TTC12.

**Table S4**. Probes sequences targeting NUP188 and PDXDC1 in MLPA. GAPDH served as a internal control.

**SUPPLEMENTARY METHODS**

**CNV analysis** All sequencing data in this study were trimmed and filtered with Fastp. The filtered sequencing reads were aligned to a hg19 reference genome (human_g1k_v37.fasta) with Burrows-Wheeler Aligner (bwa 0.7.16, http://bio-bwa.Sourceforge.net/) and the duplicates were removed with samtools markdup (samtools 1.9). Then the Genome Analysis Toolkit (gatk 4.0.12.0) was employed for base quality score recalibration and indel realignment. The common genetic variants were recalibrated using GATK Resource Bundle (dbSNP, HapMap, g1k snps and indels). We applied the popular somatic copy number alteration caller, VarScan (VarScan v2.3.9), to the pre-processed sequencing data with the workflow recommended by developers. The called copy numbers with Varscan between two samples were smoothed and segmented using the R library and DNAcopy.

**Multiplex-Ligation Dependent Probe Amplification (MLPA)** MLPA was used to validate the specific small chromosomal abnormalities at 16p13.11 identified from CNV analysis based on WE-GS data by following standard protocol. Three different probes were designed for each targeted gene (*GAPDH*, *PDXDC1* and *NUP188*). The sequences of probes were provided in Table S4. GAPDH served as internal reference. Copy number status was determined based on the DQ (dosage quotient) values (0.80<DQ<1.20, Normal; DQ=0.00, homozygous deletion; 0.40<DQ<0.65, heterozygous deletion; 1.3<DQ<1.65, heterozygous duplication; 1.75<DQ<2.15, heterozygous triplication/homozygous.
