# Supplementary material for: Biallelic mutations of TTC12 and TTC21B were identified in Chinese patients with multisystem ciliopathy syndromes
Source: Hum Genomics. 2022 Oct 22;16:48. doi: 10.1186/s40246-022-00421-z (PMC9587637; doi:10.1186/s40246-022-00421-z)
Supplement: Supplementary file 7 — Additional file 7: Table S3. Primers used for Sanger sequencing for TTC21B and TTC12 variants that identified from WES analyzes [file 40246_2022_421_MOESM7_ESM.pdf]

Table S2. Primers used for Sanger sequencing for TTC21B and TTC12 variants that identified from WES analyzes.

Primers Name	Sequences
TTC12-G542V-F	5' ATTGCTTTTGCCTCACCA 3'
TTC12-G542V-R	5' CCTTAATTCTGTTCCCCGA 3'
TTC12-c.1446-F	5' CATCCTTTGCCTGCTCTCT 3'
TTC12-c.1446-R	5' TCCAACACCCACAGAAACAG 3'
TTC12-V600-F	5' AACTGCTTGCTGTGGTTTGT 3'
TTC12-V600-R	5' TTGCTGCTAAGTTCCTCCTC 3'
TTC12-G691-F	5' TCCAGAAGCCTGAACCGT 3'
TTC12-G691-R	5' CCAGCCTAATGCCCACTC 3'
TTC21B-C518-552-F	5' TCTTCTTCCTCTTCCCTCTCA 3'
TTC21B-C518-552-R	5' TTTTCCTCCTACTGCTTGTGA 3'
TTC21B-F117-F	5' CCCCTACCAGATAGAGAAGC 3'
TTC21B-F117-R	5' AACTGGCAATAGAGTGAACAA 3'
TTC21B-c.2322-F	5' TTAGCCTGAAGAAGCCATAG 3'
TTC21B-c.2322-R	5' CCAAATAATCACCACGAAAT 3'
