# Supplementary material for: Biallelic mutations of TTC12 and TTC21B were identified in Chinese patients with multisystem ciliopathy syndromes
Source: Hum Genomics. 2022 Oct 22;16:48. doi: 10.1186/s40246-022-00421-z (PMC9587637; doi:10.1186/s40246-022-00421-z)
Supplement: Supplementary file 8 — Additional file 8: Table S4. Primers used for real-time PCR and cDNA amplification of TTC12 [file 40246_2022_421_MOESM8_ESM.pdf]

Table S3. Primers used for real-time PCR and cDNA amplification of TTC12.

Primers name	Sequences
P1:RT PCR for TTC12-F	5' CAACCACCTTGACCTGACC 3'
P1:RT PCR for TTC12-R	5' CCTCCATCCTGGCTGTTTA 3'
P2:RT PCR for TTC12-F	5' TTGGGGATGGCTGCTTG 3'
P2:RT PCR for TTC12-F	5' CACCTCCACAGCCCAAAC 3'
